# *CTLA-4* gene polymorphisms are associated with obesity in Turner Syndrome

**DOI:** 10.1590/1678-4685-GMB-2017-0312

**Published:** 2018-11-29

**Authors:** Luana Oliveira dos Santos, Adriana Valéria Sales Bispo, Juliana Vieira de Barros, Raysa Samanta Moraes Laranjeira, Rafaella do Nascimento Pinto, Jaqueline de Azevêdo Silva, Andréa de Rezende Duarte, Jacqueline Araújo, Paula Sandrin-Garcia, Sergio Crovella, Marcos André Cavalcanti Bezerra, Taciana Furtado de Mendonça Belmont, Maria do Socorro Cavalcanti, Neide Santos

**Affiliations:** ^1^ Departmento de Genética, Universidade Federal de Pernambuco, Recife, PE, Brazil; ^2^ Instituto Federal de Educação, Ciência e Tecnologia do Sertão Pernambucano, Campus Serra Talhada, Serra Talhada, PE, Brazil; ^3^ Serviço de Genética Medica, Instituto de Medicina Integral Professor Fernando Figueira, Recife, PE, Brazil; ^4^ Serviço de Endocrinlogia Pediátrica do Hospital das Clínicas, Universidade Federal de Pernambuco, Recife, PE, Brazil; ^5^ Departmento de Biofísica e Radiobiologia, Universidade Federal de Pernambuco, Recife, PE, Brazil; ^6^ Instituto de Biociências, Universidade de Pernambuco, Recife, PE, Brazil

**Keywords:** *CTLA-4* gene, immune genes, obesity, polymorphism, Turner syndrome

## Abstract

Turner syndrome (TS) is characterized by a set of clinical conditions, including autoimmune/inflammatory diseases and infectious conditions, that can compromise a patient’s quality of life. Here we assessed polymorphisms in *CTLA-4* +49A/G (rs231775)*, PTPN22 +*1858G/A (rs2476601)*,* and *MBL2 -*550 (H/L) (rs11003125), -221(X/Y) (rs7096206) and exon 1 (A/O) in women from northeastern Brazil to determine whether polymorphisms within these key immune response genes confer differential susceptibility to clinical conditions in TS. A case-control genetic association study was performed, including 86 female TS patients and 179 healthy women. An association was observed for the A/G genotype of *CTLA-4* +49A/G in TS patients (*p*=0.043, odds ratio [OR]=0.54). In addition, an association between the *CTLA-4* G/G genotype and obesity was detected in TS patients (*p*=0.02, OR=6.04). Regarding, the *-*550(H/L) polymorphism in the *MBL2* promoter, the frequency of the H/L genotype was significantly higher in the TS group than healthy controls (*p*=0.01, OR=1.96). The H/H genotype indicated a protective effect in TS patients (*p*=0.01, OR=0.23). No differences were observed in the distribution of -221(X/Y), *MBL2* exon 1 variants, and *PTPN22 +*1858G/A in any assessed groups. *CTLA-4* variants are potentially involved in obesity in this cohort of TS patients from northeastern Brazil.

## Introduction

Turner syndrome (TS) is one of the most common chromosomal abnormalities in humans and is characterized by the presence of one X chromosome and total or partial loss of the second sex chromosome. TS is estimated to affect 1 in every 2500 live female births (Stochholm *et al.*, 2006). Individuals with TS exhibit a set of phenotypic features, including short stature and gonadal dysgenesis. Other clinical conditions, such as osteoporosis, dyslipidemia, obesity and congenital malformations are also observed (Ostberg *et al.*, 2005; Carvalho *et al.*, 2010; Bispo *et al.*, 2013; Ríos Orbañanos *et al.*, 2015).

Some studies have reported increased levels of autoantibodies (anti-thyroid peroxidase and anti-glutamic-aciddecarboxylase) in TS patients and an increased risk of developing a range of autoimmune diseases, such as Hashimoto’s thyroiditis, type I diabetes mellitus, celiac disease, Crohn’s disease, ulcerative colitis, juvenile rheumatoid arthritis, Addison’s disease, autoimmune hepatitis, psoriasis, vitiligo, and alopecia (Mortensen *et al.*, 2009; Bianco *et al.*, 2010; Jørgensen *et al.*, 2010; Bakalov *et al.*, 2012). In addition, ovarian insufficiency and absence of a second normal X chromosome are linked to an increased risk of autoimmune disorders in these patients. However, the underlying pathophysiological mechanisms related to the immune unbalance remain to be fully elucidated (Mortensen *et al.*, 2009; Bakalov *et al.*, 2012).

A wide variety of autoimmune/inflammatory diseases and infectious conditions have been associated with a set of genes related to immune regulation, including the *tyrosine-protein phosphatase non-receptor type 22 gene* (*PTPN22),* cytotoxic T-lymphocyte-associated protein 4 gene (*CTLA4),* and mannose-binding lectin *(MBL2)* (Bottini *et al.*, 2004; Bevilacqua Filho *et al.*, 2012; Katkam *et al.*, 2015). Polymorphisms within these three genes have been analyzed due to their importance in immune balance and homeostasis within the body. Even though a body of evidence indicates immune deregulation processes in TS, only *PTPN22* rs2476601 has been assessed in Brazilian TS patients from São Paulo (Southeast region) (Bianco *et al.*, 2010). Furthermore, studies evaluating the role of *MBL2* and *CTLA-4* polymorphisms in TS and its association with clinical features are lacking.

To understand the role of these key genes in immune misbalance and its consequences, we assessed whether *PTPN22*, *CTLA-4,* and *MBL2* polymorphisms confer susceptibility to autoimmune conditions or other inflammation-related features in TS patients from Northeast Brazil.

## Material and Methods

### Patients and controls

This study included 86 patients with cytogenetic diagnosis of TS, who attended at Medical Genetics Service of Institute of Integral Medicine Professor Fernando Figueira and at Pediatric Endocrinology Service of Clinical Hospital of Federal University of Pernambuco. It was proposed as a pilot study. At time of TS diagnosis, patients mean age was 11.48 years old (SD ± 7.52 years old), ranging from 0.1 to 33 years. Clinical data shown in the Table 1 were obtained from medical records of each patient. The control group included 179 healthy women from the same geographical region. Their mean age was 34.62 years old (SD ± 13.4 years old), ranging from 8 to 72 years. Exclusion criteria for the control group included an individuals’ history of autoimmune and inflammatory chronic disease, also in close relatives such as parents. All individuals (or their legal responsible) included in this research signed an informed consent term, which followed the Declaration of Helsinki guidelines and presented the approval number from local Ethics Committee (Record: CEP/IMIP N° 802/06; CEP/CCS/UFPE N° 493/11).

**Table 1:** Clinical characterization of all Turner syndrome patients enrolled in our study.

Clinical characteristics	N
Short stature	69
Skeletal abnormalities	72^a^
Osteopenia/osteoporosis	4
Sexual infantilism	100^b^
Primary amenorrhea	31
Obesity	9
Dyslipidemia	5
Autoimmune thyroid disease	11
Alopecia	2
Inflammatory diseases	9
Infectious diseases	6
Neurological disease	23
Cardiovascular disease	17
Renal malformations	10
Skin diseases	17
Edema	35
Mammary hypertelorism	22
Hearing impairment	7
Ear malformations	21
Muscle hypotonia	3
Arched palate	7
Nails malformations	33
Eyes anatomic alterations	13
Visual impairment	2
Short and webbed neck	35
Low posterior hairline	21
Skin redundancy in the neck	18

^a^ Main clinical conditions are *cubitus valgus*, *pectus scavatum* and *genu valgus*;

^b^ Absent mammary development, absent axillary and pubic hair, hypoplastic uterus and absent ovaries;

### Karyotyping

Clinical diagnosis of 86 TS patients was confirmed by chromosome analysis in peripheral blood leucocyte. Karyotypes found were as follows: 45,X (n=47, 54.65%); 45,X/46,X,i(Xq) (n=15, 17.44%); 46,X,i(Xq) (n=4, 4.65%); 45,X/46,XY (n=4, 4.65%); 45,X/46,X,r(X) (n=3, 3.49); other chromosomal constitutions summed 13 individuals (15.12%).

### DNA extraction and genotyping

Genomic DNA was extracted from whole blood using IllustraTM Blood GenomicPrep Mini Spin Kit (GE Healthcare) according to manufacturer’s instructions. SNPs selection was based on minimum allele frequency (MAF) of 10% and/or SNP consequence/function upon gene action. A total of five SNPs were selected distributed as follows *PTPN22* + 1858G/A (rs2476601) at codon 620, *CTLA-4* +49A/G (rs231775) within codon 17 in the first exon and *MBL2* promoter region -550(H/L) (rs11003125), -221(X/Y) (rs7096206). Genotyping was performed using TaqMan SNP genotyping assays and Taqman Universal Master Mix (Applied Biosystems®, CA) according to manufacturer instructions. SNP assessment within *MBL2* exon 1 (A/O) was performed using SYBR Green (Qiagen, Hilden, Germany) as previously described (Hladnik *et al.*, 2002). All Sybr Green endpoint PCRs were performed, including all three possible genotypes as positive controls, in a Rotor-Gene 6000 TM apparatus (Corbett Research Mortlake, Sydney, Australia). Ten randomly chosen *MBL2* genotyped samples were sequenced in order to double-check the Melting Temperature assay (MTA) results. We found 100% concordance between sequenced samples and the MTA results.

### Statistical analysis

Statistical analyses were carried out using SNPStats available at http://bioinfo.iconcologia.net/SNPstats_web and R software (https://www.r-project.org/). Hardy–Weinberg equilibrium was tested for each polymorphism by comparing observed with expected frequencies using chi-square (χ2) tests. Differences in allele and genotype frequencies from each studied polymorphism in patients and controls were assessed using χ^2^ or Fisher’s exact test. Odds Ratio (OR) and 95% Confidence Intervals (CI) were also calculated and a *p*-value < 0.05 was considered statistically significant. Combined alleles for the *PTPN22* and *CTLA-4* genes, haplotypes for the *MBL2* gene and a possible association of these combined alleles and haplotype with clinical conditions in TS patients were also assessed. Combined genotypes for the *MBL2* gene were assessed by Arlequin version 3.1 software (Excoffier *et al.*, 2005). Fisher’s exact test was performed to evaluate difference between combined genotypes in case-control and associations with clinical data in TS group. We compared genotype and allele distribution for all SNPs assessed in this study in TS patients and control group. Posteriorly, we evaluated a possible association of all polymorphic variants with differential presence of autoimmune diseases such as autoimmune thyroid disease and alopecia and other clinical features as follows: obesity, dyslipidemia, inflammatory and infectious conditions in TS patients. Post-hoc (goodness of fit χ^2^ tests) power analysis was performed with the G* Power software (version 3.1.9.2, available at http://www.gpower.hhu.de/), with α error probability of 0.05

## Results

### *PTPN22* and *CTLA-4* gene polymorphisms

The allele and genotype distributions of *PTPN22* rs2476601 (G > A) and *CTLA-4* rs231775 (A > G) among TS patients and healthy controls are summarized in Table 2. Conformity to Hardy-Weinberg equilibrium (HWE; *p* > 0.05) was observed in both SNP distributions, and no significant differences were found in the allele and genotype frequencies of these variants in both groups (Table 2).

**Table 2:** Genotype and allele distribution of *PTPN22* and *CTLA-4* gene polymorphisms in TS and controls group.

Polymorphism	Patients N (%)	Controls N (%)	Odds ratio (95% CI)	*p*-value
***PTPN22*** rs2476601				
Allele	172	358		
G	170 (99%)	345 (96%)	1.00	
A	2 (1%)	13 (4%)	0.31 (0.03-1.40)	0.16
Genotype	86	179		
GG	84 (97.7%)	166 (92.7%)	1.00	
GA	2 (2.3%)	13 (7.3%)	0.30 (0.03-1.39)	0.15
AA	0 (0.0%)	0 (0.0%)	0 (0 inf)	1.00
***CTLA-4*** rs231775				
Allele	172	340		
A	114 (66%)	203 (60%)	1.00	
G	58 (34%)	137 (40%)	0.75 (0.50-1.12)	0.17
Genotype	86	170		
AA	41 (47.7%)	59 (34.7%)	1.00	
AG	32 (37.2%)	85 (50%)	0.54 (0.29 - 0.99)	0.043*
GG	13 (15.1%)	26 (15.3%)	0.72 (0.30-1.66)	0.44

TS = Turner syndrome; CI = confidence interval; *p* < 0.05 was considered statistically significant.

^*^ Significant difference using Fisher’s exact test.

We did not detect the homozygous A/A genotype of *PTPN22* rs2476601 (G > A) in any of the studied groups. A lower frequency of the A allele and G/A genotype was observed in both TS patients and controls (Table 2). No significant association was identified between the assessed SNPs and the presence of any clinical conditions in women with TS (*p* > 0.05; Fisher’s exact test) (Table S1). The power was 80.7% (α-error = 5% confidence) to detect a medium effect size (w=0.3) for *PTPN22* genotypes in both patients and controls.

Concerning *CTLA-4* rs231775, significantly different distributions of genotype frequencies were observed in TS patients compared to the control group. An association was detected for the A/G genotype (*p*=0.043, OR=0.54), indicating a differential distribution for this SNP in TS patients. The power was 99% (α-error = 5% confidence) to detect a medium effect size (w=0.3) for *CTLA4* genotypes comparing patients and controls. Furthermore, when assessing the clinical features of TS and the allele and genotype distribution, we detected an association between the *CTLA-4* allele (recessive model: A/A—A/G vs*.* G/G) and obesity in TS patients (*p*=0.02, 95% CI 1.37-26.75, OR=6.04) (Table 3, Table S2).

**Table 3:** Genotype distribution of *CTLA-4* gene polymorphisms in TS group.

Model	Polymorphism	Obesity N (%)	Non-obesity N (%)	Odds ratio (95% CI)	*p*-value
Recessive	***CTLA-4***				
	rs231775				
	Genotype				
	AA- AG	5 (55.6%)	68 (88.3%)	1.00	
					
	GG	4 (44.4%)	9 (11.7%)	6.04 (1.37 - 26.75)	**0.023**

CI = confidence interval; *p* < 0.05 was considered statistically significant.

### Combined alleles for *PTPN22* and *CTLA-4* genes

The combined alleles of *PTPN22* and *CTLA-4* and frequencies for both groups are given in Table 5. No significant differences were observed. Furthermore, no significant association was established between the combined alleles and clinical status of TS patients (Table S3).

**Table 4:** Genotype and allele distribution of *MBL2* gene polymorphisms in TS and controls group.

Inheritance Model	Polymorphism	Patients N (%)	Controls N (%)	Odds ratio (95% CI)	*p*-value
	***MBL2*** **rs11003125**				
	Allele	172	300		
	L	116 (67.0%)	198 (66.0%)	1.00	
	H	56 (33.0%)	102 (34.0%)	0.93 (0.61- 1.42)	0.76
Recessive	Genotype	86	150		
	L/L—H/L	83 (96.5%)	130 (86.7%)	1.00	
	H/H	3 (3.5%)	20 (13.3%)	0.23 (0.04-0.83)	0.01^*^
Overdominant	Genotype	86	150		
	L/L—H/H	36 (41.9%)	88 (58.7%)	1.00	
	H/L	50 (58.1%)	62 (41.3%)	1.96 (1.11-3.50)	0.01^*^
	***MBL2*** **rs7096206**				
	Allele	172	300		
	Y	147 (85%)	256 (85%)	1.00	
	X	25 (15%)	44 (15%)	0.98(0.55-1.73)	1.0
Recessive	Genotype	86	150		
	Y/Y—X/Y	84 (97.7%)	144 (96.0%)	1.00	
	X/X	2 (2.3%)	6 (4.0%)	0.57(0.05-3.29)	0.7
	***MBL2*** **Exon 1**				
	Allele	126	300		
	A	98 (78%)	253 (84%)	1.00	
	O	28 (22%)	47 (16%)	1.53(0.87-2.66)	0.12
Dominant	Genotype	63	150		
	A/A	39 (61.9%)	108 (72%)	1.00	
	A/O - O/O	24 (38.1%)	42 (28%)	1.57(0.80-3.06)	0.14

TS = Turner syndrome; CI = confidence interval; *p* < 0.05 was considered statistically significant.

^*^ Significant difference using Fisher’s exact test.

**Table 5:** Analyses of combined alleles of *PTPN22* and *CTLA4* genes, haplotypes and genotypes of -550 and -221 promoter region and exon 1 of *MBL2* gene in TS patients and controls.

*PTPN22*	*CTLA4*	Frequencies in TS	Frequencies in controls	*p*-value	OR (95% C.I.)
G	A	0.6512	0.5697	Reference	1.00
G	G	0.3372	0.3939	0.16	0.76 (0.52-1.11)
A	A	0.0116	0.027	0.25	0.39 (0.08 - 1.93)
A	G	0	0.0093	1	0.00 (-Inf - Inf)
Global haplotype association *p*-value: 0.14					
MBL producers and haplotype		Frequencies in TS	Frequencies in controls	*p*-value	OR (95% C.I.)
High MBL producers					
LYA		0.3827	0.4272	Reference	1.00
HYA		0.2586	0.2694	0.89	1.04 (0.62 1.73)
Low MBL producers					
LXA		0.1453	0.1467	0.69	1.12 (0.64 1.94)
Deficient MBL producers					
LYO		0.1464	0.0861	0.13	1.75 (0.85 3.60)
HYO		0.0669	0.0706	0.84	1.10 (0.45 2.67)
Global haplotype association *p*-value: 0.63					
MBL producers and genotypes		TS group (n = 65)	Control (n = 150)	*p*-value	OR (95%C.I.)
High MBL producers					
HYA/HYA					
HYA/LYA		27 (0.42)	76 (0.51)	Reference	Reference
LYA/LYA					
Low MBL producers					
LXA/LXA					
LYA/LXA					
HYA/LXA					
HYA/HYO		32 (0.49)	63 (0.42)	0.278	1.42(0.74-2.76)
HYA/LYO					
LYA/LYO					
Deficient MBL producers					
HYO/LXA					
HYO/LYO					
LYO/LXA		6 (0.09)	11 (0.07)	0.558	1.53(0.42-5.06)
LYO/LYO					
HYO/HYO					

### *MBL2* gene polymorphisms

The *MBL2* genotype and allele distributions in TS patients and controls are given in Table 4. All allelic and genotypic frequencies of *MBL2* polymorphisms were in HWE in the TS and control groups, except for rs11003125 (-550 H/L) in the TS group (*p* < 0.05). The H/L genotype frequency was significantly higher in TS patients than controls (overdominant model: H/L vs. L/L—H/H: *p*=0.01, OR=1.96, 95% CI 1.11-3.50). Furthermore, the H/H genotype indicated a protective effect in TS patients compared to healthy controls (recessive model: H/H vs. L/L—H/L: *p*=0.01, OR=0.23, 95% CI 0.04-0.83). Again, this result cannot be fully explained due to an absence of HWE in this specific group. The power was 99.9% (5% confidence) to detect a medium effect size (w=0.3) for -550 *MBL2* genotypes in both groups.

Regarding *MBL2* rs7096206 (-221 *X/Y*), no differences in allele or genotype frequencies were observed between TS patients and controls. The power was 30.5% (5% confidence) to detect a medium effect size (w=0.3) for -221 *MBL2* genotypes when comparing patients and controls. Furthermore, no significant differences were observed in the genotype and allele frequencies of exon 1 variants between TS patients and controls. The power was 91.2% (5% confidence) to detect a medium effect size (w=0.3) for exon 1 *MBL2* genotypes when comparing TS patients and controls. In patients with TS, no SNP in the *MBL2* gene or promotor region was associated with clinical characteristics (Table S4).

### Haplotypes and combined genotypes of *MBL2* gene *-550, -221,* and exon 1 variants

The frequencies of haplotypes and combined genotypes originating from linkage disequilibrium between the *MBL2* -550 and -221 promoter region and exon 1 polymorphisms are given in Table 5. Haplotypes were combined in different groups; haplotypes associated with high expression of MBL (LYA, HYA), low production of MBL (LXA), and deficient expression of MBL (LYO, HYO). No significant differences were found in haplotype frequencies between the analyzed groups. Furthermore, no significant association was established among haplotypes and the clinical data of TS patients (Table S5).

Genotypes were classified as high (HYA/HYA, HYA/LYA, and LYA/LYA); low (LXA/LXA, LYA/LXA, HYA/LXA, HYA/HYO, HYA/LYO, and LYA/LYO); and deficient producers of MBL (HYO/HYO, HYO/LXA, HYO/LYO, LYO/LXA, and LYO/LYO). Significant differences were not found between the evaluated groups. Moreover, no significant difference was observed among combined genotypes and the clinical data of TS patients (Table S6).

## Discussion

To date, only a few assays have been performed involving genes linked to innate and adaptive immunity in patients with TS, even though the immune response seems impaired in these patients (Bianco *et al.*, 2010, 2012).

We included *PTPN22* in our analyses due to its importance in the host immune system; this gene encodes LYP, an important negative regulator of T cell activation (Bottini *et al.*, 2004). Our results indicate an absence of an association between the selected *PTPN22* SNP and autoimmunity, inflammatory, and infectious conditions in TS women.

Our results differ from Bianco *et al.* (2010), who found an association between the same SNP (*PTPN22* rs2476601) and the development of autoimmune diseases in TS in another cohort from Southeast Brazil. The frequency of the A allele in women with TS (1.1%) and healthy controls (3.6%), as well as the heterozygote genotype in both groups (2.3% and 7.3%, respectively), was lower in our study than that of Bianco *et al.* (2010). In their study, the frequency of the A allele and heterozygote genotype were 18.3% and 28.2% in the TS group, and 9.2% and 16.1% in controls, respectively. A disease-associated homozygote genotype was present in 4.2% of patients and 1.1% of controls.

This difference between studies could be due to variation in allele frequencies of some disease-associated SNPs in different ethnic groups (Mori *et al.*, 2005), because the Brazilian population exhibits variety in allele distribution. Geographic distributions in Brazil exhibit ethnic disparities, mainly due to the genetic burden of heterogeneous colonization sources (Pena *et al.*, 2009; Coelho *et al.*, 2015).

We also evaluated *PTPN22* rs2476601 (G > A) and other clinical features in TS patients, such as obesity, as well as dyslipidemia, given its role in modulating inflammatory conditions. As obesity is a disease characterized by chronic mild inflammation, the concentration of acute phase proteins and cytokines associated with inflammation are higher in obese individuals compared to normal weight individuals (Trayhurn, 2007). However, our analysis did not indicate an association between these clinical manifestations in the TS group, which is similar to a previous study by Salinas-Santander *et al.* (2016), in which *PTPN22* +1858G/A was not associated with differential susceptibility to overweight and the development of obesity in adolescents.

The *CTLA-4* +49A/G polymorphism at exon 1 is involved in the negative regulation of T cells. Our assay showed an association between the rs231775 G/G genotype and obesity in the TS group (*p*=0.02).

The presence of the *CTLA-4* +49A/G variant has been associated with different diseases, and G/G individuals may possess CTLA-4 protein with a weak suppression function compared to individuals with the A/A genotype (Chistiakov and Turakulov, 2003). Therefore, increased T-cell activation due to this reduced inhibitory signal to T cells would be associated with the pathogenesis of several autoimmune/inflammatory diseases, including obesity, as observed with TS patients in the present study. The adipocytes of obese individuals with TS express fewer anti-inflammatory elements and high amounts of pro-inflammatory factors, leading to a misplaced response in immune cells (Ostberg *et al.*, 2005; Bakalov *et al.*, 2012).

*MBL2* polymorphisms and their influence on serum protein levels have been evaluated extensively and found to be associated with recurrent and severe infections (Sumiya *et al.*, 1991; Summerfield *et al.*, 1995), such as tuberculosis (da Cruz *et al.*, 2013) and autoimmune diseases, including celiac disease (Boniotto *et al.*, 2005), systemic lupus erythematosus (Lee *et al.*, 2005), Sjögren’s syndrome (Tsutsumi *et al.*, 2001), and autoimmune thyroid disease (AITD) (Bevilacqua Filho *et al.*, 2012).

Polymorphisms at exon 1 and the promotor region of *MBL2* were evaluated in our study due to their role in innate immunity and modulation of the inflammatory response. A relationship between clinical parameters and this genetic variant has not been assessed previously in TS, making our study the first to be performed in women with TS.

No association was found between the *MBL2* -221 (*X/Y* allele) polymorphism and different clinical conditions in TS patients. This *MBL2* promoter variation has a significant down-regulating effect on the serum MBL concentration, leading to ineffective clearance of apoptotic cells and the spread of self-antigens, permitting an immune response toward autoimmunity and tissue damage (Bouwman *et al.*, 2006; Araujo *et al.*, 2009). The *Y* variant is associated with high serum MBL expression (Madsen *et al.*, 1995) and has been involved in susceptibility to the development of different diseases (Lee *et al.*, 2005; da Cruz *et al.*, 2013).

On the other hand, our results revealed significant differences regarding the -550 (H/L allele) promoter polymorphism between TS patients and controls (*p* < 0.05), revealing a different distribution in both groups.

Notably, in our study population, the -550 H/L *MBL2* variant was not in HWE, though the power analysis excluded type I and II statistical error. Therefore, we suggest that the TS condition acts upon the allelic distribution, causing deviation from HWE. Although differences in the *MBL2* polymorphism distribution have been detected, no significant association was found regarding the *MBL2* -550 (H/L allele) variant and clinical data of the TS group.

In summary, even though our study is a pilot study, due to limited number of TS patients and controls included, our results indicate a differential distribution for some polymorphisms within key inflammation-regulating genes in TS patients. The understanding of how key immune genes and its variants are related in TS might help in future therapy strategies. These findings may open up a new potential line of research to improve life’s quality in these individuals.

## Supplementary material

The following online material is available for this article:

Table S1 - Detailed statistics results for *PTPN22* rs2476601 (G/A)

Table S2 - Detailed statistics results for *CTLA-4* rs231775 (A/G).

Table S3 - Detailed statistics results for Combined Allelexs.

Table S4 - Detailed statistics results for the *MBL2* gene and promotor region

Table S5 - Detailed statistics results for the haplotypes (LYA, HYA, LXA, LYO, HYO) of the *MBL2* gene.

Table S6 - Detailed statistics results for the genotypes (HighMBL expression, Intermediate MBL expression and Low MBL expression) of the *MBL2* gene.
