# Supplementary material for: CTLA-4 gene polymorphisms are associated with obesity in Turner Syndrome
Source: Genet Mol Biol. 2018 Nov 29;41(4):727–34. doi: 10.1590/1678-4685-GMB-2017-0312 (PMC6415610; doi:10.1590/1678-4685-GMB-2017-0312)
Supplement: Supplementary file 1 [file 1415-4757-GMB-1678-4685-GMB-2017-0312-s001.pdf]

Supplementary Material to "CTLA-4 gene polymorphisms are associated with obesity in Turner Syndrome"

Table S1 - Results for chi-square and Fisher's test concerning *PTPN22* rs2476601 (G/A), for those not shown within the main manuscript text. In Results we wrote "No significant association was identified between the assessed SNP and the presence of any clinical conditions in women with TS (data not shown)." Below are these data in the tables:

Clinical conditions: autoimmune thyroid disease - Genotype distribution of *PTPN22* gene polymorphisms in TS group (n=86).

Model	Polymorphism	TS patients without autoimmune thyroid disease N (%)	TS patients with Autoimmune thyroid disease N (%)	Odds ratio (95% CI)	*p*-value
Recessive	***PTPN22*** rs2476601 Genotype				
	GG	73 (97.3%)	11 (100%)	1.00	
	AG	2 (2.7%)	0 (0%)	0.00 (0.00-NA)	0.46

CI = Confidence Intervals

Fisher's exact test: *p*-value = 1

Clinical condition: alopecia - Genotype distribution of *PTPN22* gene olymorphisms in TS group (n=86).

Model	Polymorphism	Without alopecia N (%)	Alopecia N (%)	Odds ratio (95% CI)	*p*-value
Recessive	***PTPN22*** rs2476601 Genotype				
	GG	82 (97.6%)	2 (100%)	1.00	
	AG	2 (2.4%)	0 (0%)	0.00 (0.00-NA)	0.76

CI = Confidence Intervals

Fisher's exact test: *p*-value = 1

Clinical condition: obesity - Genotype distribution of *PTPN22* gene polymorphisms in TS group (n=86).

Model	Polymorphism	Non-obesity N (%)	Obesity N (%)	Odds ratio (95% CI)	*p*-value
Recessive	***PTPN22*** rs2476601 Genotype				
	GG	75 (97.4%)	9 (100%)	1.00	
	AG	2 (2.6%)	0 (0%)	0.00 (0.00-NA)	0.5

CI = Confidence Intervals

Fisher's exact test: *p*-value = 1

Clinical condition: dyslipidemia - Genotype distribution of *PTPN22* gene polymorphisms in TS group (n=86)

Model	Polymorphism	Without dyslipidemia N (%)	Dyslipidemia N (%)	Odds ratio (95% CI)	*p*-value
Recessive	***PTPN22*** rs2476601 Genotype				
	GG	79 (97.5%)	5 (100%)	1.00	
	AG	2 (2.5%)	0 (0%)	0.00 (0.00-NA)	0.62

CI = confidence interval

Fisher's exact test: *p*-value = 1

Clinical condition: inflammatory conditions - Genotype distribution of *PTPN22* gene polymorphisms in TS group (n=86).

Model	Polymorphism	Without inflammatory conditions N (%)	Inflammatory conditions N (%)	Odds ratio (95% CI)	*p*-value
Recessive	*PTPN22* rs2476601 Genotype				
	GG	75 (97.4%)	9 (100%)	1.00	
	AG	2 (2.6%)	0 (0%)	0.00 (0.00-NA)	0.5

CI = Confidence Intervals

Fishers's exact test *p*-value = 1

Clinical condition: infectious conditions - Genotype distribution of *PTPN22* gene polymorphisms in TS group (n=86).

Model	Polymorphism	Without infectious conditions N (%)	Infectious Conditions N (%)	Odds ratio (95% CI)	*p*-value
Recessive	*PTPN22* rs2476601 Genotype				
	GG	78 (97.5%)	6 (100%)	1.00	
	AG	2 (2.6%)	0 (0%)	0.00 (0.00-NA)	0.5

OR = Odds Ratio; CI = Confidence Intervals

Fisher's exact test: *p*-value = 1
