# Supplementary material for: CTLA-4 gene polymorphisms are associated with obesity in Turner Syndrome
Source: Genet Mol Biol. 2018 Nov 29;41(4):727–34. doi: 10.1590/1678-4685-GMB-2017-0312 (PMC6415610; doi:10.1590/1678-4685-GMB-2017-0312)
Supplement: Supplementary file 2 [file 1415-4757-GMB-1678-4685-GMB-2017-0312-s002.pdf]

Supplementary Material to "CTLA-4 gene polymorphisms are associated with obesity in Turner Syndrome"

Table S2 - Results for chi-square and Fisher's test concerning concerning *CTLA-4* rs231775 (A/G). The association between the *CTLA-4* variant and obesity is described in results of the manuscript: (recessive model: A/A-A/G vs. G/G) and obesity in TS patients (p = 0.02, 95% CI 1.37 - 26.75, OR=6.04). In relation to the others clinical data, no significant association was identified between the assessed SNP and the presence of any clinical conditions in women with TS. Below are these data in tables.

Clinical conditions: autoimmune thyroid disease - Genotype distribution of *CTlA-4* gene polymorphisms in TS group (n=86).

Model	Polymorphism	TS patients without autoimmune thyroid disease N (%)	TS patients with Autoimmune thyroid disease N (%)	Odds ratio (95% CI)	*p*-value
Codominant	*CTLA-4* rs231775 Genotype				
	AA	35 (46.7%)	6 (54.5%)	1.00	
	AG[1]	28 (37.3%)	4 (36.4%)	0.83 (0.213.24)	0.79
	GG[2]	12 (16%)	1 (9.1%)	0.49 (0.054.46)	

CI = Confidence Intervals

[1] Fisher's exact test: *p*-value = 1

[2] Fisher's exact test: *p*-value = 1

Clinical condition: alopecia - Genotype distribution of *CTlA-4* gene polymorphisms in TS group (n=86).

Model	Polymorphism	Without alopecia N (%)	Alopecia N (%)	Odds ratio (95% CI)	*p*-value
Codominant	*CTLA-4* rs231775 Genotype				
	AA	41 (48.8%)	0 (0%)	1.00	
	AG[1]	30 (35.7%)	2 (100%)	NA (0.00 - NA)	0.13
	GG[2]	13 (15.5%)	0 (0%)	1.00 (0.00 - NA)	

CI = Confidence Intervals

[1] Fusher's exact test: *p*-value = 0.1

[2] Fisher's exact test: *p*-value = 1

Clinical condition: dyslipidemia - Genotype distribution of *CTlA-4* gene polymorphisms in TS group (n=86).

Model	Polymorphism	Without	Dyslipidemia	Odds ratio (95% CI)	*p*-value

		dyslipidemia N (%)	N (%)		
Codominant	*CTLA-4* rs231775 Genotype				
	AA	39 (48.1%)	2 (40%)	1.00	
	AG[1]	31 (38.3%)	1 (20%)	0.63 (0.05-7.26)	0.35
	GG[2]	11 (13.6%)	2 (40%)	3.55 (0.45-28.13)	

CI = Confidence Intervals

[1] Fisher's exact test: *p*-value = 1

[2] Fisher's exact test: *p*-value = 0.2

Clinical condition: inflammatory conditions - Genotype distribution of *CTlA-4* gene polymorphisms in TS group (n=86).

Model	Polymorphism	Without inflammatory conditions N (%)	Inflammatory conditions N (%)	Odds ratio (95% CI)	*p*-value
Codominant	*CTLA-4* rs231775 Genotype				
	AA	35 (45.5%)	6 (66.7%)	1.00	
	AG[1]	31 (40.3%)	1 (11.1%)	0.19 (0.021.65)	0.18
	GG[2]	11 (14.3%)	2 (22.2%)	1.06 (0.196.03)	

CI = Confidence Intervals

[1] Fisher's exact test: *p*-value = 0.12

[2] Fisher's exact test: *p*-value = 1

Clinical condition: infectious conditions - Genotype distribution of *CTlA-4* gene polymorphisms in TS group (n=86).

Model	Polymorphism	Without infectious conditions N (%)	Infectious conditions N (%)	Odds ratio (95% CI)	*p*-value
Codominant	*CTLA-4* rs231775 Genotype				
	AA	37 (46.2%)	4 (66.7%)	1.00	
	AG[1]	30 (37.5%)	2 (33.3%)	0.62 (0.113.60)	0.31
	GG[2]	13 (16.2%)	0 (0%)	0.00 (0.00-NA)	

CI = Confidence Intervals

[1] Fisher's exact test: *p*-value = 0.6

[2] Fisher's exact test: *p*-value = 0.5
