# Supplementary material for: CTLA-4 gene polymorphisms are associated with obesity in Turner Syndrome
Source: Genet Mol Biol. 2018 Nov 29;41(4):727–34. doi: 10.1590/1678-4685-GMB-2017-0312 (PMC6415610; doi:10.1590/1678-4685-GMB-2017-0312)
Supplement: Supplementary file 3 [file 1415-4757-GMB-1678-4685-GMB-2017-0312-s003.pdf]

Supplementary Material to "CTLA-4 gene polymorphisms are associated with obesity in Turner Syndrome"

Table S3 - Results concerning Combined Alleles. In Results we wrote: “No significant association was established between the combined alleles and clinical status of TS patients (data not shown).” Below are the data in tables.

Clinical conditions: autoimmune thyroid disease - Analyses of Combined Alleles of *PTPN22* and *CTLA4* gene in TS patients (n=86)

PTPN22 gene	*CTLA4* gene	Frequencies in TS without autoimmune thyroid disease	Frequencies in TS with autoimmune thyroid disease	*p* - value	OR (95% C.I.)
G	A	0.64	0.7273	Reference	1.00
G	G	0.3467	0.2727	0.52	0.74 (0.29- 1.86)
A	A	0.0133	NA	1	0.00 (-Inf - Inf)
A	G	0	NA	0.00 (Inf - Inf)	0.00 (-Inf - Inf)
Global haplotype association *p*- value: 0.61					

OR = Odds Ratio; CI = Confidence Intervals

Clinical condition: alopecia - Analyses of Combined Alleles of *PTPN22* and *CTLA4* gene in TS patients (n=86)

PTPN22 gene	*CTLA4* gene	Frequencies in TS without alopecia	Frequencies in TS with alopecia	*p* - value	OR (95% C.I.)
G	A	0.6549	0.5	Reference	1.00
G	G	0.3332	0.5	0.54	1.78 (0.29 -11.06)
A	A	0.0118	NA	1	0.00 (-Inf - Inf)
A	G	0	NA	0.00 (Inf - Inf)	0.00 (-Inf - Inf)
Global haplotype association *p*- value: 0.79					

OR = Odds Ratio; CI = Confidence Intervals

Clinical condition: obesity - Analyses of Combined Alleles of *PTPN22* and *CTLA4* gene in TS patients (n=86)

PTPN22 gene	*CTLA4* gene	Frequencies in non-obesity TS	Frequencies in obesity TS	*p* - value	OR (95% C.I.)
G	A	0.6764	0.4444	Reference	1.00
G	G	0.3106	0.5556	0.073	2.37 (0.93 -6.03)
A	A	0.0119	NA	1	0.00 (-Inf - Inf)
A	G	0.001	NA	0.00 (Inf - Inf)	0.00 (-Inf - Inf)
Global haplotype association *p*- value: 0.15					

OR = Odds Ratio; CI = Confidence Intervals

Clinical condition: dyslipidemia - Analyses of Combined Alleles of *PTPN22* and *CTLA4* gene in TS patients (n=86)

PTPN22 gene	*CTLA4* gene	Frequencies in TS patients without dyslipidemia	Frequencies in TS patients With dyslipidemia	*p* - value	OR (95% C.I.)
G	A	0.6608	0.5	Reference	1.00
G	G	0.3269	0.5	0.32	1.83 (0.56 -5.98)
A	A	0.0121	NA	1	0.00 (-Inf - Inf)
A	G	3e04	NA	0.00 (Inf - Inf)	0.00 (-Inf - Inf)
Global haplotype association *p*- value: 0.54					

OR = Odds Ratio; CI = Confidence Intervals

Clinical condition: inflammatory conditions - Analyses of Combined Alleles of *PTPN22* and *CTLA4* gene in TS patients (n=86)

PTPN22 gene	*CTLA4* gene	Frequencies in TS patients without inflammatory conditions	Frequencies in TS patients With inflammatory conditions	*p* - value	OR (95% C.I.)
G	A	0.6429	0.7222	Reference	1.00
G	G	0.3441	0.2778	0.59	0.76 (0.28- 2.08)
A	A	0.013	NA	1	0.00 (-Inf - Inf)
A	G	0	NA	0.00 (Inf - Inf)	0.00 (-Inf - Inf)

Global haplotype association *p*- value: 0.69

OR = Odds Ratio; CI = Confidence Interval

Clinical condition: infectious conditions - Analyses of Combined Alleles of *PTPN22* and *CTLA4* gene in TS patients (n=86)

PTPN22 gene	*CTLA4* gene	Frequencies in TS patients without infectious conditions	Frequencies in TS patients With infectious conditions	*p* - value	OR (95% C.I.)
G	A	0.6375	0.8333	Reference	1.00
G	G	0.35	0.1667	0.25	0.42 (0.10 1.82)
A	A	0.0125	NA	0.00 (Inf - Inf)	0.00 (-Inf - Inf)
A	G	0	NA	0.00 (Inf - Inf)	0.00 (-Inf - Inf)

OR = Odds Ratio; CI = Confidence Intervals
