# Supplementary material for: CTLA-4 gene polymorphisms are associated with obesity in Turner Syndrome
Source: Genet Mol Biol. 2018 Nov 29;41(4):727–34. doi: 10.1590/1678-4685-GMB-2017-0312 (PMC6415610; doi:10.1590/1678-4685-GMB-2017-0312)
Supplement: Supplementary file 4 [file 1415-4757-GMB-1678-4685-GMB-2017-0312-s004.pdf]

Supplementary Material to "CTLA-4 gene polymorphisms are associated with obesity in Turner Syndrome"

Table S4 – Results concerning the *MBL2* gene and promotor region

1. In results section of the manuscript, we wrote: "In patients with TS, no SNP in the *MBL2* gene or promotor region was associated with clinical characteristics (data not shown)." Below are the data in tables

Clinical conditions: autoimmune thyroid disease - The genotype distribution of the *MBL2* gene polymorphisms in TS.

Model	Polymorphism	TS patients without autoimmune thyroid disease N (%)	TS patients with autoimmune thyroid disease N (%)	Odds ratio (95% CI)	*p*-value
	MBL2 rs11003125				
Recessive	Genotype				
	L/L-H/L	73 (97.3%)	10 (90.9%)	1.00	0.35
	H/H	2 (2.7%)	1 (9.1%)	3.65 (0.304-4.02)	
	MBL2 rs7096206				
Recessive	Genotype				
	Y/Y-X/Y	73 (97.3%)	11 (100%)	1.00	0.46
	X/X	2 (2.7%)	0 (0%)	0.00 (0.00-NA)	
	MBL2 Exon 1				
Dominant	Genotype				
	A/A	33 (58.9%)	6 (85.7%)	1.00	0.14
	A/O - O/O	23 (41.1%)	1 (14.3%)	0.24 (0.03-2.12)	

CI = confidence interval

(*MBL2* rs11003125): using Fisher's exact test: *p*-value = 0.3
(*MBL2* rs7096206): using Fisher;s exact test: *p*-value = 1
(*MBL2* Exon 1): using Fisher's exact test: *p*-value = 0.2

Clinical condition: alopecia - The genotype distribution of the *MBL2* gene polymorphisms in TS

Model	Polymorphism	TS patients without alopecia N (%)	TS patients with alopecia N (%)	Odds ratio (95% CI)	*p*-value
	MBL2 rs11003125				
Recessive	Genotype				
	L/L-H/L	81 (96.4%)	2 (100%)	1.00	0.7
	H/H	3 (3.6%)	0 (0%)	0.00 (0.00-NA)	
	MBL2 rs7096206				
Recessive	Genotype				
	Y/Y-X/Y	82 (97.6%)	2 (100%)	1.00	0.76
	X/X	2 (2.4%)	0 (0%)	0.00 (0.00NA)	
	MBL2 Exon 1				
Dominant	Genotype				
	A/A	39 (62.9%)	0 (0%)	1.00	0.16
	A/O - O/O	23 (37.1%)	1 (100%)	NA (0.00NA)	

CI = confidence interval

(*MBL2* rs11003125): Fisher's exact test: *p*-value = 1
(*MBL2* rs7096206): Fisher's exact test: *p*-value = 1
(*MBL2* Exon 1): Fisher's exact test: *p*-value =0.3

Clinical condition: obesity - The genotype distribution of the *MBL2* gene polymorphisms in TS

Model	Polymorphism	Non-obesity N (%)	Obesity N (%)	Odds ratio (95% CI)	*p*-value
	MBL2 rs11003125				
Recessive	Genotype				
	L/L-H/L	74 (96.1%)	9 (100%)	1.00	0.7
	H/H	3 (3.9%)	0 (0%)	0.00 (0.00-NA)	
	MBL2 rs7096206				
Recessive	Genotype				
	Y/Y-X/Y	75 (97.4%)	9 (100%)	1.00	0.5

	X/X	2 (2.6%)	0 (0%)	0.00 (0.00-NA)	
	MBL2 Exon 1				
Dominant	Genotype				
	A/A	35 (63.6%)	4 (50%)	1.00	0.46
	A/O - O/O	20 (36.4%)	4 (50%)	1.75 (0.39-7.77)	

CI = confidence interval

(*MBL2* rs11003125): Fisher's exact test: *p*-value = 1
(*MBL2* rs7096206): Fisher's exact test:*p*-value = 1
(*MBL2* Exon 1): Fisher's exact test: *p*-value = 0.4

Clinical condition: dyslipidemia - The genotype distribution of the *MBL2* gene olymorphisms in TS.

Model	Polymorphism	Without dyslipidemia N (%)	With dyslipidemia N (%)	Odds ratio (95% CI)	*p*-value
	MBL2 rs11003125				
Recessive	Genotype				
	L/L-H/L	79 (97.5%)	4 (80%)	1.00	0.13
	H/H	2 (2.5%)	1 (20%)	9.87 (0.73133.24)	
	MBL2 rs7096206				
Recessive	Genotype				
	Y/Y-X/Y	80 (98.8%)	4 (80%)	1.00	0.073
	X/X	1 (1.2%)	1 (20%)	20.00 (1.05381.40)	
	MBL2 Exon 1				
Dominant	Genotype				
	A/A	37 (61.7%)	2 (66.7%)	1.00	0.86
	A/O - O/O	23 (38.3%)	1 (33.3%)	0.80 (0.079.38)	

CI = confidence interval

(*MBL2* rs11003125): Fisher's exact test: *p*-value =0.1
(*MBL2* rs7096206): Fisher's exact test:*p*-value = 0.1
(*MBL2* Exon 1): Fisher's exact test: *p*-value = 1

Clinical condition: inflammatory conditions - The genotype distribution of the *MBL2* gene polymorphisms in ST.

Model	Polymorphism	Without inflammatory conditions N (%)	With inflammatory conditions N (%)	Odds ratio (95% CI)	*p*-value
	MBL2 rs11003125				
Recessive	Genotype				
	L/L-H/L	74 (96.1%)	9 (100%)	1.00	0.41
	H/H	3 (3.9%)	0 (0%)	0.00 (0.00-NA)	
	MBL2 rs7096206				
Recessive	Genotype				
	Y/Y-X/Y	76 (98.7%)	8 (88.9%)	1.00	0.15
	X/X	1 (1.3%)	1 (11.1%)	9.50 (0.54-166.88)	
	MBL2 Exon 1				
Dominant	Genotype				
	A/A	34 (60.7%)	5 (71.4%)	1.00	0.58
	A/O - O/O	22 (39.3%)	2 (28.6%)	0.62 (0.11-3.47)	

CI = confidence interval

MBL2 rs11003125): Fisher's exact test: *p*-value = 1
(*MBL2* rs7096206): Fisher's exact test: *p*-value = 0.1
(*MBL2* Exon 1): Fisher's exact test: *p*-value = 0.6

Clinical condition: infectious conditions - The genotype distribution of the *MBL2* gene polymorphisms in ST.

Model	Polymorphism	Without infections conditions N (%)	With infections conditions N (%)	Odds ratio (95% CI)	*p*-value
	MBL2 rs11003125				
Recessive	Genotype				
	L/L-H/L	78 (97.5%)	5 (83.3%)	1.00	0.17
	H/H	2 (2.5%)	1 (16.7%)	7.80 (0.60-101.42)	
	MBL2 rs7096206				
Recessive	Genotype				
	Y/Y-X/Y	79 (98.8%)	5 (83.3%)	1.00	0.092
	X/X	1 (1.2%)	1 (16.7%)	15.80 (0.86-291.64)	
	MBL2 Exon 1				
Dominant	Genotype				
	A/A	35 (59.3%)	4 (100%)	1.00	0.045
	A/O - O/O	24 (40.7%)	0 (0%)	0.00 (0.00-NA)	

CI = confidence interval

(*MBL2* rs11003125): Fisher's exac test: *p*-value = 0.1
(*MBL2* rs7096206): Fisher's exac test:*p*-value = 0.1
(*MBL2* Exon 1): Fisher's exac test:*p*-value = 0.2
