# Supplementary material for: CTLA-4 gene polymorphisms are associated with obesity in Turner Syndrome
Source: Genet Mol Biol. 2018 Nov 29;41(4):727–34. doi: 10.1590/1678-4685-GMB-2017-0312 (PMC6415610; doi:10.1590/1678-4685-GMB-2017-0312)
Supplement: Supplementary file 5 [file 1415-4757-GMB-1678-4685-GMB-2017-0312-s005.pdf]

Supplementary Material to "CTLA-4 gene polymorphisms are associated with obesity in Turner Syndrome"

Table S5 – Results concerning the haplotypes (LYA, HYA, LXA, LYO, HYO) of the *MBL2* gene. In Results section of the manuscript, we wrote: “No significant association was established among haplotypes (LYA, HYA, LXA, LYO, HYO) and the clinical data of TS patients (data not shown).” Below are the data in tables.

Clinical conditions: autoimmune thyroid disease

Analyses of Haplotypes of the -550 and -221 promoter region and exon 1 of the *MBL2* gene in TS patients

MBL expression and Haplotype	Frequencies in TS Patients without autoimmune thyroid disease	Frequencies in TS patients with autoimmune thyroid disease	*p* - value	OR (95% C.I.)
High MBL expession				
LYA	0.3764	0.4863	Reference	1.00
HYA	0.247	0.3011	0.93	1.06 (0.29-3.82)
Intermediate MBL expression				
LXA	0.1466	0.1193	0.67	0.74 (0.19- 2.92)
Low MBL expression				
LYO	0.1503	0.0763	0.48	0.45 (0.05- 4.05)
HYO	0.0796	0	0.84	1.10 (0.45 - 2.67)

OR = Odds Ratio; CI = Confidence Intervals

Clinical condition: obesity

Analyses of Haplotypes of the -550 and -221 promoter region and exon 1 of the *MBL2* gene in TS patients

MBL expression and Haplotype	Non-obesity	Obesity	*p* - value	OR (95% C.I.)
High MBL expession				
LYA	0.3748	0.4596	Reference	1.00
HYA	0.2774	0.1111	0.41	0.51 (0.10 - 2.48)
Intermediate MBL expression				
LXA	0.1493	-	0.74	0.75 (0.15- 3.86)
Low MBL expression.				
LYO	0.1447	0.2626	0.82	1.20 (0.26- 5.55)
HYO	0.0538	0.0556	0.54	1.89 (0.25- 14.11)

Global haplotype association p - value: 0.75

OR = Odds Ratio; CI = Confidence Intervals

Clinical condition: dyslipidemia

Analyses of Haplotypes of the -550 and -221 promoter region and exon 1 of the *MBL2* gene in TS patients

MBL expression and Haplotype	Frequencies in TS Patients without dyslipidemia	Frequencies in TS patients with dyslipidemia	*p* - value	OR (95% C.I.)
High MBL expession				
LYA	0.41	0.45	Reference	1.00
HYA	0.2298	0.2	0.48	0.50 (0.07-3.37)
Intermediate MBL expression				
LXA	0.1064	0.1	0.72	1.36 (0.25-7.44)
Low MBL expression.				
LYO	0.1502	0.15	0.94	0.92 (0.09- 9.14)
HYO	0.068	NA	1	0.00 (Inf-Inf)
Global haplotype association p - value: 0.68				

OR = Odds Ratio; CI = Confidence Intervals

Clinical condition: inflammatory conditions

MBL expression and Haplotype	Frequencies in TS Patients without inflammatory conditions	Frequencies in TS patients with inflammatory conditions	*p* - value	OR (95% C.I.)
High MBL expession				
LYA	0.3602	0.4861	Reference	1.00
HYA	0.2823	0.2222	0.21	0.37 (0.08- 1.73)
Intermediate MBL expression				
LXA	0.1428	0	0.83	0.85 (0.21- 3.47)
Low MBL expression.				
LYO	0.1658	0.0694	0.28	0.29 (0.03- 2.72)
HYO	0.0489	0.0556	0.59	1.82 (0.21- 15.89)

Global haplotype association p - value: 0.52

OR = Odds Ratio; CI = Confidence Intervals

Analyses of Haplotypes of the -550 and -221 promoter region and exon 1 of the *MBL2* gene in TS patients

Clinical condition: infectious conditions

Analyses of Haplotypes of the -550 and -221 promoter region and exon 1 of the *MBL2* gene in TS patients

MBL expression and Haplotype	Frequencies in TS Patients without infectious conditions	Frequencies in TS patients with infectious conditions	*p* - value	OR (95% C.I.)
High MBL expession				
LYA	0.4232	0.3333	Reference	1.00
HYA	0.2095	0.4167	0.29	3.01 (0.39-23.18)
Intermediate MBL expression				
LXA	0.0954	0.25	-	-
Low MBL expression.				
LYO	0.1627	NA	-	-
HYO	0.0672	NA	-	-

OR = Odds Ratio; CI = Confidence Intervals
