# Supplementary material for: CTLA-4 gene polymorphisms are associated with obesity in Turner Syndrome
Source: Genet Mol Biol. 2018 Nov 29;41(4):727–34. doi: 10.1590/1678-4685-GMB-2017-0312 (PMC6415610; doi:10.1590/1678-4685-GMB-2017-0312)
Supplement: Supplementary file 6 [file 1415-4757-GMB-1678-4685-GMB-2017-0312-s006.pdf]

Supplementary Material to "CTLA-4 gene polymorphisms are associated with obesity in Turner Syndrome"

Table S6 – Results concerning to the genotypes (High MBL expression, Intermediate MBL expression and Low MBL expression): MBL2 gene. In Results we wrote: “No significant difference was observed among combined genotypes (high, low deficient producers of MBL) and the clinical data of TS patients (data not shown).” Fisher’s exact test was performed to evaluate the possible associations with clinical data in TS group. Below are the data in tables.

Clinical conditions: autoimmune thyroid disease

Analyses of Combined Genotypes of the -550 and -221 promoter region and exon 1 of the *MBL2* gene in TS patients (n=65)

MBL expression and Genotypes	TS patients without autoimmune thyroid disease N	TS patients with autoimmune thyroid disease N	*p* - value	OR (95% C.I.)
Intermediate MBL expression				
**LXA/LXA**<br>**LYA/LXA**<br>**HYA/LXA**<br>**HYA/HYO**<br>**HYA/LYO**<br>**LYA/LYO**	30	2	Reference	Reference
High MBL expression				
**HYA/HYA**<br>**HYA/LYA**<br>**LYA/LYA**	23	4	0.39	2.56 (0.33-30.70)
Low MBL expression				
**HYO/HYO**<br>**HYO/LXA**<br>**HYO/LYO**<br>**LYO/LXA**<br>**LYO/LYO**	6	0	1	0 (0.00 - 30.09)

OR = Odds Ratio; CI = Confidence Intervals

Clinical condition: alopecia

Analyses of Combined Genotypes of the -550 and -221 promoter region and exon 1 of the *MBL2* gene in TS patients (n=65)

MBL expression and Genotypes	TS patients without Alopecia N	TS patients with Alopecia disease N	*p* - value	OR (95% C.I.)
Intermediate MBL expression				
**LXA/LXA**	32	0	Reference	Reference
**LYA/LXA**				
**HYA/LXA**				
**HYA/HYO**				
**HYA/LYO**				
**LYA/LYO**				
High MBL expression.				
**HYA/HYA**	27	0	1	0 (0Inf)
**HYA/LYA**				
**LYA/LYA**				
Low MBL expression.				
**HYO/HYO**	5	1	0.1579	Inf (0.13- Inf)
**HYO/LXA**				
**HYO/LYO**				
**LYO/LXA**				
**LYO/LYO**				
**OR = Odds Ratio; CI = Confidence Intervals**				

Clinical condition: obesity

Analyses of Combined Genotypes of the -550 and -221 promoter region and exon 1 of the *MBL2* gene in TS patients (n=65)

MBL expression and Genotypes	Non-obesity TS patients N	Obesity TS patients N	*p* - value	OR (95% C.I.)
Intermediate MBL expression				
**LXA/LXA**	28	4	Reference	Reference
**LYA/LXA**				
**HYA/LXA**				
**HYA/HYO**				
**HYA/LYO**				
**LYA/LYO**				
High MBL expression.				
**HYA/HYA**	25	2	0.67	0.56(0.04-4.33)
**HYA/LYA**				
**LYA/LYA**				
Low MBL expression				
**HYO/HYO**				

MBL expression and Genotypes	Non-obesity TS patients N	Obesity TS patients N	*p* - value	OR (95% C.I.)
**HYO/LXA**	5	1	1	1.38(0.02-18.78)
**HYO/LYO**				
**LYO/LXA**				
**LYO/LYO**				
**OR = Odds Ratio; CI = Confidence Intervals**				

Clinical condition: dyslipidemia

Analyses of Combined Genotypes of the -550 and -221 promoter region and exon 1 of the *MBL2* gene in TS patients (n=65)

MBL expression and Genotypes	TS patients without dyslipidemia N	TS patients with dyslipidemia N	*p* - value	OR (95% C.I.)
Intermediate MBL expression				
**LXA/LXA**	31	1	Reference	Reference
**LYA/LXA**				
**HYA/LXA**				
**HYA/HYO**				
**HYA/LYO**				
**LYA/LYO**				
High MBL expression.				
**HYA/HYA**	24	3	0.32	3.79 (0.28-209.59)
**HYA/LYA**				
**LYA/LYA**				
Low MBL expression				
**HYO/HYO**	6	0	1	0 (0.00- 207.32)
**HYO/LXA**				
**HYO/LYO**				
**LYO/LXA**				
**LYO/LYO**				
**OR = Odds Ratio; CI = Confidence Intervals**				

Clinical condition: inflammatory conditions

Analyses of Combined Genotypes of the -550 and -221 promoter region and exon 1 of the *MBL2* gene in TS patients (n=65)

**MBL expression and Genotypes**	**TS patients without Inflammatory conditions N**	**TS patients with Inflammatory conditins N**	***p* - value**	**OR (95% C.I.)**
Intermediate MBL expression				
**LXA/LXA**	28	4	Reference	Reference
**LYA/LXA**				
**HYA/LXA**				
**HYA/HYO**				
**HYA/LYO**				
**LYA/LYO**				
High MBL expression				
**HYA/HYA**	23	4	1	1.21 (0.20-7.29)
**HYA/LYA**				
**LYA/LYA**				
Low MBL expression				
**HYO/HYO**	6	0	1	0 (0.00- 8.90)
**HYO/LXA**				
**HYO/LYO**				
**LYO/LXA**				
**LYO/LYO**				
**OR = Odds Ratio; CI = Confidence Intervals**				

Clinical condition: infectious conditions

Analyses of Combined Genotypes of the -550 and -221 promoter region and exon 1 of the *MBL2* gene in TS patients (n=65)

**MBL expression and Genotypes**	**TS patients without Infections conditions N**	**TS patients with Infectious conditins N**	***p* - value**	**OR (95% C.I.)**
Intermediate MBL expression				
**LXA/LXA**	28	4	Reference	Reference
**LYA/LXA**				
**HYA/LXA**				
**HYA/HYO**				
**HYA/LYO**				
**LYA/LYO**				
High MBL expression				
**HYA/HYA**				

MBL expression and Genotypes	TS patients without Infections conditions N	TS patients with Infectious conditins N	*p* - value	OR (95% C.I.)
**HYA/LYA**	26	1	0.365	0.27 (0.00-3.014)
**LYA/LYA**				
Low MBL expression				
**HYO/HYO**				
**HYO/LXA**				
**HYO/LYO**	6	0	1	0 (0.00- 8.90)
**LYO/LXA**				
**LYO/LYO**				
**OR = Odds Ratio; CI = Confidence Intervals**				
